# Global Typhoid Fever Incidence: A Systematic Review and Meta-analysis

**DOI:** 10.1093/cid/ciy1094

**Published:** 2019-03-07

**Authors:** Christian S Marchello, Chuen Yen Hong, John A Crump

**Affiliations:** Centre for International Health, University of Otago, New Zealand

**Keywords:** incidence studies, meta-analysis, *Salmonella enterica* serovar Typhi, typhoid fever, systematic review

## Abstract

**Background:**

Contemporary incidence estimates of typhoid fever are needed to guide policy decisions and control measures and to improve future epidemiological studies.

**Methods:**

We systematically reviewed 3 databases (Ovid Medline, PubMed, and Scopus) without restriction on age, country, language, or time for studies reporting the incidence of blood culture–confirmed typhoid fever. Outbreak, travel-associated, and passive government surveillance reports were excluded. We performed a meta-analysis using a random-effects model to calculate estimates of pooled incidence, stratifying by studies that reported the incidence of typhoid fever and those that estimated incidence by using multipliers.

**Results:**

Thirty-three studies were included in the analysis. There were 26 study sites from 16 countries reporting typhoid cases from population-based incidence studies, and 17 sites in 9 countries used multipliers to account for underascertainment in sentinel surveillance data. We identified Africa and Asia as regions with studies showing high typhoid incidence while noting considerable variation of typhoid incidence in time and place, including in consecutive years at the same location. Overall, more recent studies reported lower typhoid incidence compared to years prior to 2000. We identified variation in the criteria for collecting a blood culture, and among multiplier studies we identified a lack of a standardization for the types of multipliers being used to estimate incidence.

**Conclusions:**

Typhoid fever incidence remains high at many sites. Additional and more accurate typhoid incidence studies are needed to support country decisions about typhoid conjugate vaccine adoption. Standardization of multiplier types applied in multiplier studies is recommended.

*Salmonella enterica* subspecies *enterica* serovar Typhi (*Salmonella* Typhi) is the cause of typhoid fever. Typhoid fever is a systemic infection that is an important source of illness and death in low-resource areas [1, 2]. Persons living in areas without access to improved sanitation facilities who are exposed to fecally contaminated water and food are at greatest risk for infection [3–5]. Timely access to effective antimicrobial therapy is central to preventing complications such as intestinal perforation and death. Consequently, the alarming spread of antimicrobial resistance in *Salmonella* Typhi is likely to contribute further to poor clinical outcomes [6]. Although there has been considerable progress with expanding coverage of access to improved water and sanitation facilities in most regions except Oceania [7] and typhoid incidence has declined in some countries, typhoid remains a major problem worldwide [8]. The emergence and spread of antimicrobial resistance in *Salmonella* Typhi and the long-term efforts needed to deliver water and sanitation improvements has focused increasing attention on the use of typhoid vaccines.

Two vaccines, Vi polysaccharide (Vi-PS) and live attenuated oral vaccine (Ty21a), have been widely licensed for typhoid prevention [9]. However, their use is restricted to those aged ≥2 years for Vi-PS and ≥6 years for Ty21a [10]. This, combined with waning immunity after 2 years of vaccination [11–13], means that Vi-PS and Ty21a are not widely used in typhoid-endemic areas. Typhoid conjugate vaccines (TCVs) overcome a number of limitations of Vi-PS and Ty21a, having been shown to be safe, immunogenic, and effective in infants and young children [14–16]. In October 2017, the World Health Organization (WHO) Strategic Advisory Group of Experts on Immunization recommended TCV for routine use in children >6 months of age in typhoid-endemic countries. In December 2017, the first TCV was prequalified by WHO [17], enabling typhoid-endemic, low-income countries priority access and funding for the vaccine [18].

Contemporary estimates of the incidence of typhoid fever are helpful in supporting countries’ decision making about typhoid prevention, including vaccine use. While there have been improvements in the sophistication of efforts to model typhoid fever incidence [2, 19, 20], primary data have not always been at the forefront. Recognizing a recent increase in the number of typhoid fever incidence studies [8], we performed a systematic review of the literature and a meta-analysis. Our goal was to describe, summarize, and analyze high-quality primary incidence data on typhoid fever. We focused primarily on prospective studies reporting incidence either through population-based surveillance or studies estimating incidence through sentinel site surveillance with a healthcare utilization survey to establish multipliers to account for underascertainment.

## METHODS

### Search Strategy

We performed a systematic review of articles published in 3 databases: Ovid, Scopus, and PubMed. We searched Ovid Medline from 1946 to 19 January 2018 with Daily Update, Embase Classic + Embase, and EBM Reviews–Cochrane Central Register of Controlled Trials. Scopus and PubMed databases were searched from inception to 22 January 2018 (search strategies in Supplementary Appendix A). Additionally, reference lists of any article included after the full-text review were searched.

Duplicates from the 3 searches were removed and collated using an online systematic review tool [21]. The resulting list of titles and abstracts were independently screened by 2 authors (J. A. C. and C. Y. H.) for relevance. Any article selected by at least 1 reviewer was moved to full-text review. All subsequent processes of the systematic review were performed in parallel by 2 authors (C. S. M. and J. A. C.), with discrepancies resolved by discussion. The Preferred Reporting Items for Systematic Reviews and Meta-Analyses (PRISMA) checklist was used to document the search process [22]. Full texts of the articles were screened for inclusion. After the final list of included articles was established, data for study characteristics and incidence were abstracted.

### Inclusion and Exclusion Criteria

Epidemiologic studies of any design reporting the incidence of typhoid fever were included. No restrictions were placed on age of population, country, language, or date. For intervention studies such as controlled field trials, we used only the control and unvaccinated arms. Only cases confirmed through blood or bone marrow culture were included. We excluded studies where we were unable to separate blood or bone marrow culture-confirmed typhoid from the results of serology (eg, Widal test) or other culture sources (eg, stool, urine).

We excluded studies that were outbreak-associated, case reports, or of travel-associated typhoid fever. In addition, editorials, commentaries, and conference presentations or abstracts were excluded. While government surveillance reports (eg, US FoodNet) have valuable data and likely provide very accurate estimates, countries with robust national surveillance systems experience low typhoid incidence, and typhoid fever in these countries is often predominantly travel-associated. The combination of low incidence with the problem of distinguishing travel-associated from non-travel-associated disease underpinned the decision to exclude such reports from this systematic review. Finally, we excluded studies reporting typhoid incidence in special subsets of populations such as only human immunodeficiency virus–infected persons, and healthcare-associated or hospital-acquired infections.

### Data Abstraction and Analysis

Study characteristics that were abstracted included PubMed identifier if available, first author, publication year, study design, city or region and country of study, sample size (total population of surveillance or size of control/unvaccinated group), number of cases of fever, number of blood cultures collected, inclusion age and range, and criteria for blood culture. We subsequently classified study sites by their United Nations geographic areas and regions [23].

The currently available tools for assessing the quality and risk of bias did not translate to disease prevalence or incidence reviews [24, 25]. Therefore, we developed criteria specifically for this review, using established quality assessment tools as guidelines (Supplementary Appendix B). Our goal was to evaluate the overall quality of the study in providing an accurate estimate of typhoid incidence in the study setting. We applied greater weight to questions about study design, patient selection, and criteria for collecting a blood culture. Our form evaluated selection and performance and the reference standard, assigning “low,” “moderate,” “high,” “uncertain,” “inapplicable,” “yes,” or “no” to each question. We then assigned an overall score of low, moderate, or high quality to the study.

Data for incidence were abstracted in Excel 2016 (Microsoft, Redmond, Washington). We stratified studies into 2 main types. Studies that estimated incidence through sentinel site surveillance with multipliers to account for underascertainment were categorized as multiplier studies [26]. All other studies were categorized as population-based, which included active household or population-based surveillance, prospective observational studies, and randomized vaccine field trials with control arms. We noted the year the observation period began and how many months the surveillance followed participants.

We recorded the number of typhoid cases that occurred during the study period and then divided by the number of months the study conducted surveillance and multiplied by 12 to report incidence as a rate of cases per 100 000 per year. For multiplier studies, we recorded the estimated incidence separately and also noted the multipliers that were used.

Pooled incidence estimates were calculated with MetaXL version 5.3 software (Epigear International) using a random-effects model [27]. As a secondary analysis of published data this study was exempt from institutional review board approval.

## RESULTS

**Figure 1.**
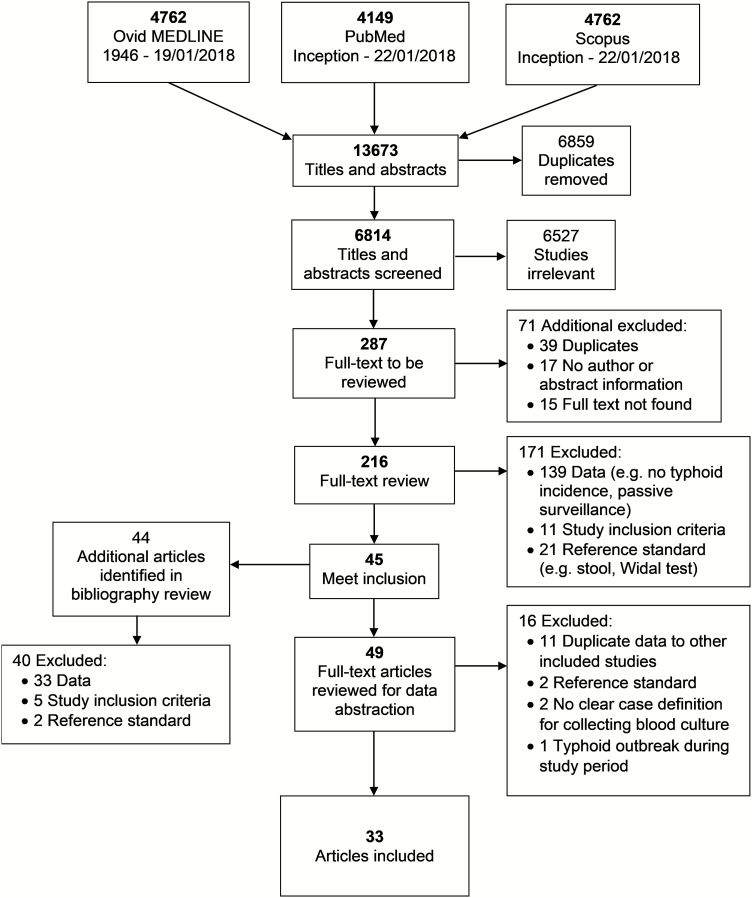
Preferred Reporting Items for Systematic Reviews and Meta-Analyses (PRISMA) flow diagram of search strategy and selection of articles, systematic review and meta-analysis of global typhoid incidence, 1946–2018.

Our search returned 13 673 results; after removing 6859 duplicates, a total of 6814 titles and abstracts were reviewed (Figure 1). We eliminated 6527 studies as irrelevant, 39 as additional duplicates, and 32 that we were unable to locate. Two hundred sixteen full-text articles were reviewed; of those, we included 49. After initial data abstraction of study characteristics, 16 additional studies were excluded: 11 contained duplicate data to other included studies [28–38], 2 used an inappropriate reference standard [39, 40], 2 did not have clear criteria for blood culture collection [41, 42], and 1 was conducted during a typhoid outbreak [43]. A total of 33 articles were included in the analysis [11–13, 15, 44–72].

### Study Characteristics and Quality Assessment

Of the 33 included articles, 17 (51.5%) were vaccine field trials, 7 (21.2%) household or population-based active surveillance studies, 4 (12.1%) sentinel surveillance, 3 (9.1%) prospective observational studies, and 2 (6.1%) of mixed study design (Table 1). Eight were categorized as multiplier studies [45, 48, 49, 56–58, 65, 68], estimating incidence with multipliers to account for underascertainment, while the remaining 25 were population based.

**Table 1.** Characteristics of Included Studies of Typhoid Fever Incidence by United Nations Classification of Geographic Region, Global, 1954–2018

United Nations Area, Region	Country [Reference]	Study Design	Years of Data Collection	Population Under Surveillance	Inclusion Age	Criteria for Blood Culture
Africa	13 sites in 10 African countries^a^ [56]	Sentinel surveillance with multipliers (12 sites); active surveillance study, household-based (1 site)^b,c^	2010–2014	7574 to 466 737	Asante Akim North <15 y; other sites all ages	≥37.5°C
Eastern Africa	Kenya [45]	Active surveillance, population-based^c^	2007–2009	28 000	All ages	≥38.0°C of any length, respiratory illness
	Kenya [45]	Active surveillance, population-based^c^	2006–2009	25 000	All ages	≥38.0°C of any length, respiratory illness, hospitalized
	Tanzania [68]	Prospective observational^c^	2009–2010	500 600	>2 mo	≥37.5°C or history of fever
Middle Africa	…	…	…	…	…	…
Northern Africa	Egypt [69]	Vaccine trial^b^	1978–1981	15 902	6–7 y	Fever (undefined) >3 d
	Egypt [48]	Sentinel surveillance with multipliers^c^	2001	664 000	≥6 mo	Fever (undefined) ≥3 d
	Egypt [65]	Sentinel surveillance with multipliers^c^	2002	2 238 590	≥1 y	≥38.0°C for >2 d or clinical diagnosis of typhoid
Southern Africa	South Africa [52]	Vaccine trial^b^	1985–1987	11 691	5–16 y	Children missing school for ≥3 d with ≥37.5°C
	South Africa [53]	Vaccine trial^b^	1985–1988	Not provided	5–16 y	Children missing school for ≥3 d with ≥37.5°C
Western Africa	Burkina Faso [49]	Sentinel surveillance with multipliers^c^	2013–2014	Not provided	2 mo–15 y	≥37.5°C for 2 d; ≤35.5°C, suspected severe local infection
	Ghana [58]	Sentinel surveillance with multipliers^c^	2007–2009	5333	<5 y	All admissions to pediatric ward
Asia						
	Indonesia, Vietnam, China, Pakistan, India [59]	Prospective observational (Indonesia, Vietnam, China); Active surveillance, household-based (Pakistan, India)^b^	2001–2004	41 845 to 97 928	China: 5–60 y; India, Jakarta, Indonesia: all ages; Pakistan 2–15 y; Vietnam: 5–18 y	Fever (undefined) ≥3 d
Eastern Asia	China [70]	Vaccine trial^b^	1994–1995	40 388	5–55 y	Suspected cases
	China [71]	Vaccine trial^b^	1995–1996	65 984	3–50 y	>38°C for >1 d
Central Asia	…	…	…	…	…	…
South-Eastern Asia	Indonesia [63]	Vaccine trial^b^	1986–1989	10 268	3–44 y	Fever (undefined) ≥3 d
	Indonesia [61]	Prospective observational^b^	2001–2003	160 261	All ages	Fever (undefined) ≥3 d
	Vietnam [55]	Prospective observational^b^	1995–1996	28 329	All ages	≥38.5°C for ≥3 d
	Vietnam [54]	Vaccine trial^b^	1998–2000	5566	2–5 y	≥37.5°C for ≥3 d
Southern Asia	Bangladesh [46]	Active surveillance, population-based^b^	2000–2001	889	All ages	Age ≥5 y: ≥37.8°Cfor ≥3 d; age <5 y: ≥37.8°C any length
	Bangladesh [57]	Active surveillance, population-based^c^	2003–2004	24 893	All ages	Age ≥5 y: ≥37.8°Cfor ≥3 d; age <5 y: ≥37.8°C any length
	India [47]	Vaccine trial^b^	1974	7292	6–17 y	Fever (undefined) ≥3 d
	India [64]	Active surveillance, household-based^b^	1995–1996	7159	<40 y	Age >5 y: ≥38°Cfor ≥3 d; age ≤5 y: ≥38°C
	India [67]	Active surveillance, household-based^b^	2004	60 452	All ages	Fever (undefined) ≥3 d
	India [66]	Vaccine trial^b^	2004–2006	18 804	≥2 y	Fever (undefined) ≥3 d
	India [15]	Vaccine trial^b^	2012–2013	860	6 mo–12 y	Fever (undefined) >3 d
	Nepal [44]	Vaccine trial^b^	1986–1987	3450	5–44 y	≥37.8°C for ≥3 d
	Pakistan [62]	Active surveillance, household-based^b^	1999–2001	11 668	<16 y	Fever (undefined) ≥5 d then ≥3 d
	Pakistan [60]	Active surveillance, household-based^b^	2007–2008	5570	<5 y	≥38.0°C
	Pakistan [11]	Vaccine trial^b^	2002–2007	13 993	2–16 y	Fever (undefined) ≥3 d
Western Asia	…	…	…	…	…	…
Europe						
Eastern Europe	Russia [50]	Vaccine trial^b^	1961–1964	91 425	≥7 y	Feverish illness lasting >3 d
	Russia [51]	Vaccine trial^b^	1966–1967	70 855	7–20 y	Feverish illness lasting >3 d
Northern Europe	…	…	…	…	…	…
Southern Europe	Yugoslavia [72]	Vaccine trial^b^	1954–1960	11 988	5–50 y	Fever (undefined)
Western Europe	…	…	…	…	…	…
Americas						
Caribbean	…	…	…	…	…	…
Central America	…	…	…	…	…	…
South America	Chile [13]	Vaccine trial^b^	1982–1987	27 305	5–22 y	Suspected cases
	Chile [12]	Vaccine trial^b^	1983–1986	21 906	5–19 y	Suspected cases
	Chile [12]	Vaccine trial^b^	1986–1991	10 302	5–19 y	Suspected cases
Northern America	…	…	…	…	…	…
Oceania	…	…	…	…	…	…

Abbreviations: y, years; mo, months; d, days; C, Celsius. ^a^Sites and countries included: Nioko and Polesgo, Burkina Faso; Bandim, Guinea-Bissau; Pikine, Senegal; Asante Akim North, Ghana; East Wad Medani, Sudan; Butajira, Ethiopia; Imerintsiatosika and Isotry, Madagascar; Pietermaritzburg, South Africa; Moshi Urban and Moshi Rural Districts, Tanzania; Kibera, Kenya.

^b^Population-based study.

^c^Multiplier study.

Among included studies, data collection was conducted from 1954 through 2014, with 17 (51.5%) of the studies collecting data from 2000 through 2014. With respect to location, studies were from 21 countries, including 6 (18.2%) from India, 4 (12.1%) from Pakistan, 3 (9.1%) from Egypt, and 3 (9.1%) from Indonesia. United Nations regions lacking any study data were Middle Africa, Central and Western Asia, Northern and Western Europe, the Caribbean, Central America, Northern America, and Oceania. The participant population age ranged down to 2 months. The median (range) size of the total population under surveillance or size of the control or unvaccinated group in vaccine trials by study was 28822 (860–2 238 590).

Through our quality assessment, we determined that 5 of the included studies were of high quality [44, 45, 55, 57, 64]. These studies did not place any restrictions on inclusion age, gave clear and explicit temperature and duration for collecting blood culture, and actively searched the community for cases. We rated 12 studies as moderate quality [46, 48, 49, 56, 59–61, 65–68, 71], and the remaining 16 as lower quality. Multiplier studies were discounted compared with population-based surveillance studies on the basis of lack of validation of the “multiplier method,” and assumed shortcomings of accuracy.

### Typhoid Incidence Among Population-based Studies

Population-based typhoid incidence studies were available in 26 sites from 16 countries (Supplementary Table 1). There were a total of 50 separate estimates of typhoid incidence; 7 studies provided the number of cases for each separate year of surveillance during the study period [12, 13, 50, 51, 53, 69, 72]. The majority of estimates of typhoid incidence at all sites were older; data for 34 (68.0%) were collected prior to the year 2000. The overall pooled estimate of incidence was 154.0 (95% confidence interval [CI], 115.1–198.6) typhoid cases per 100 000 per year.

**Figure 2.**
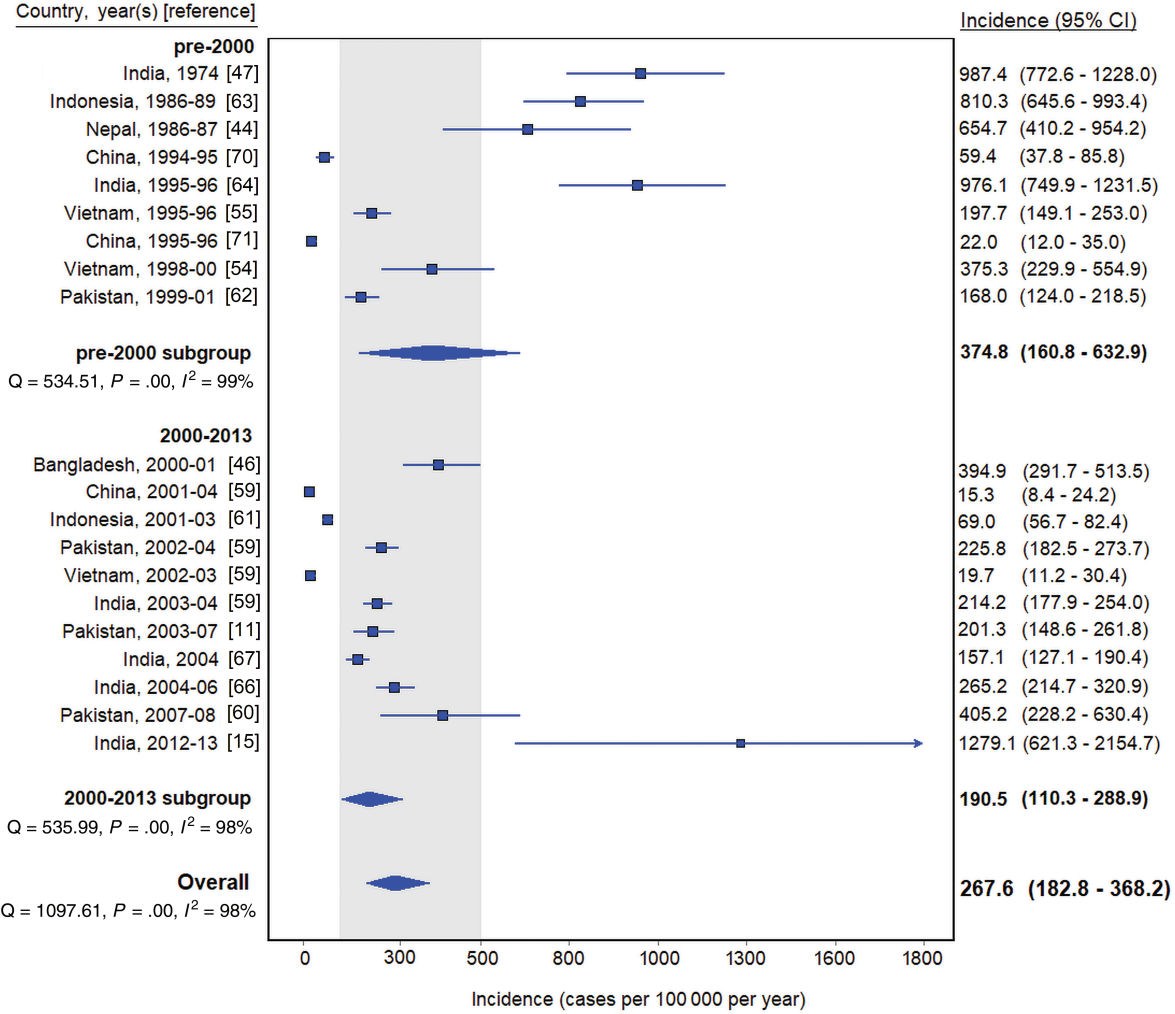
Typhoid incidence estimates among population-based studies in Asia, 1954–2018. Gray shading indicates 100–500 per 100 000 per year. Abbreviation: CI, confidence interval.

**Figure 3.**
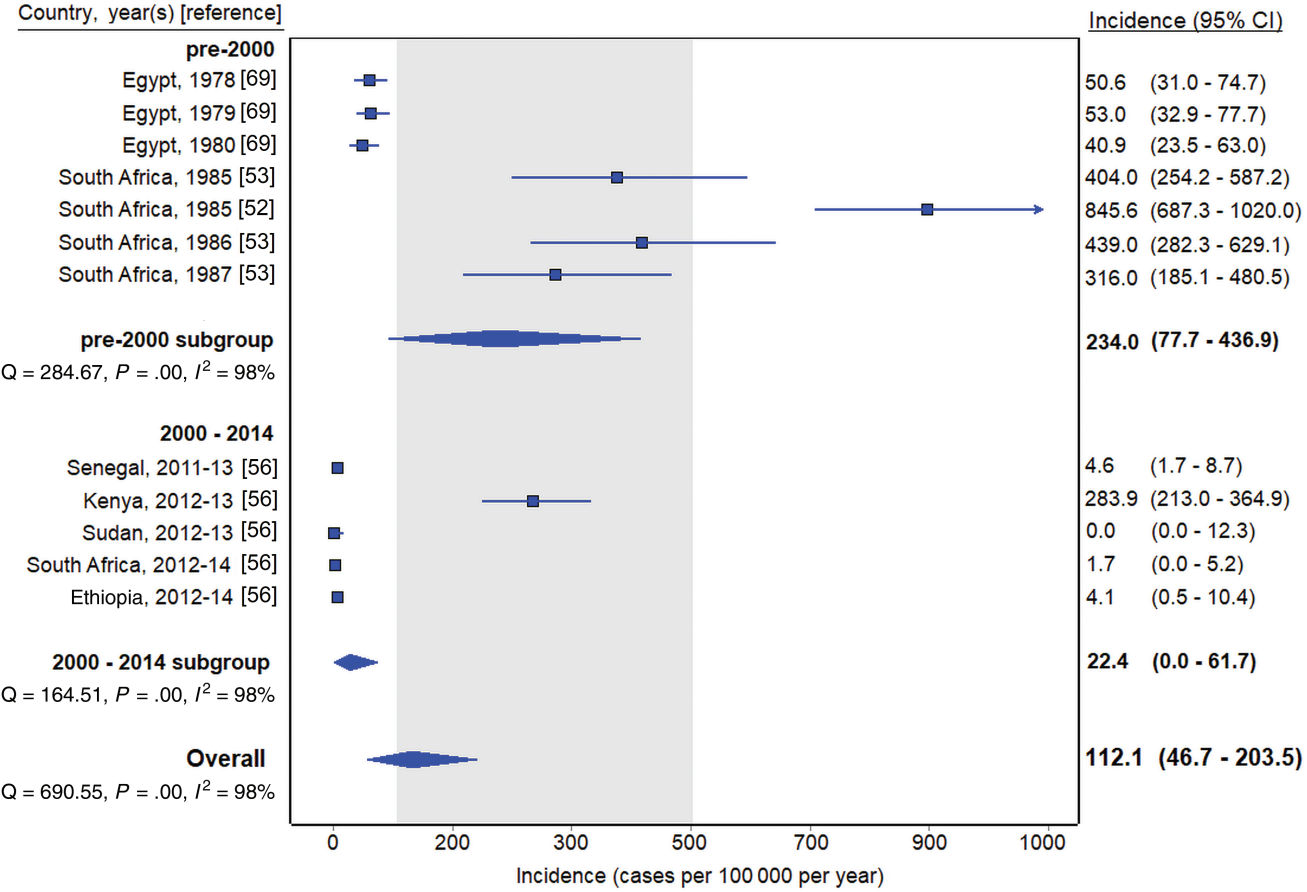
Typhoid incidence estimates among population-based studies in Africa, 1954–2018. Gray shading indicates 100–500 per 100 000 per year. Abbreviation: CI, confidence interval.

Estimates from South America (9 [18.0%]) and Eastern and Southern Europe (9 [18.0%]) had data collection periods from 1982 through 1991 and 1954 through 1967, respectively. The remaining 32 (64.0%) estimates were from the Asia or Africa geographical region. In study sites located only in Asia, the pooled incidence estimate was 267.6 (95% CI, 182.8–368.2) typhoid cases per 100 000 per year (Figure 2). Six (30.0%) of the sites in the Asia region were from India. The pooled incidence among sites located only in India was 497.2 (95% CI, 291.9–754.8) typhoid cases per 100 000 per year. Two of the Indian studies were from 1974 and 1995 [47, 64], and data were collected from the remaining 4 from 2003 through 2012 [15, 59, 66, 67]. For estimates located only in Africa, the pooled incidence estimate was 112.1 (95% CI, 46.7–203.5) typhoid cases per 100 000 per year (Figure 3). All pooled estimates had significant heterogeneity (*I*^2^ > 97%; *P* < .001, Cochran Q test).

### Typhoid Incidence Among Multiplier Studies

Studies that used a multiplier to estimate typhoid incidence were reported in 17 sites in 9 countries (Table 2). Among these studies, 4 types of multipliers were implemented:

**Table 2.** Characteristics of Multiplier Studies of Typhoid Fever Incidence, Global, 1954–2018

United Nations Area, Region	Site, Country	Year Observation Began	Adjusted Incidence Cases/100 000/Year	Age-Specific Adjusted Incidence Cases/100 000/Year	Multipliers^a^
Africa					
Eastern Africa	Lwak, Kenya [45]	2006	445.0^b^	0–1 y: 345.7^b^ 2–4 y: 742.6^b^ 5–9 y: 215.5^b^ 10–17 y: 260.4^b^ 18–34 y: 815.7^b^ 35–49 y: 0.0^b^ ≥50 y: 565.8^b^	1, 2
	Kibera, Kenya [45]	2007	822.0^b^	0–1 y: 821.5^b^ 2–4 y: 2242.6^b^ 5–9 y: 1788.0^b^ 10–17 y: 869.9^b^ 18–34 y: 312.1^b^ 35–49 y: 100.0^b^ ≥50 y: 66.6^b^	1, 2
	Pemba Island, Tanzania [68]	2010	110	≤5 y: 84 5–15 y: 101 >15 y: 128	1, 2, 3
	Imerintsiatosika, Madagascar [56]	2011	58^b^	0–1 y: 0 2–4 y: 0 5–14 y: 171 <15 y: 95 ≥15 y: 20 All: 58	1, 2
	Moshi Urban District, Tanzania [56]	2011	168^b^	0–1 y: 0 2–4 y: 1028 5–14 y: 103 <15 y: 155 ≥15 y: 201 All: 168	1, 2
	Moshi Rural District, Tanzania [56]	2011	20^b^	0–1 y: 0 2–4 y: 0 5–14 y: 18 <15 y: 18 ≥15 y: 28 All: 20	1, 2
	Isotry, Madagascar [56]	2012	42^b^	0–1 y: 0 2–4 y: 0 5–14 y: 62 <15 y: 42 ≥15 y: 42 All: 42	1, 2
Northern Africa	Bilbeis, Egypt [48]	2001	12.60	…	1, 3, 4
	Fayoum, Egypt [65]	2002	59	0–4 y: 6 5–9 y: 143 10–14 y: 160 ≥15 y: 34	1, 3
	East Wad Medani, Sudan [56]	2012	0	0–1 y: 0 2–4 y: 0 5–14 y: 0 <15 y: 0 ≥15 y: 0 All: 0	1, 2
	Ashanti, Ghana [58]	2007	330	…	1, 2
	Asante Akim North, Ghana [56]	2010	389^b^	0–1 y: 120 2–4 y: 1079 5–14 y: 314 <15 y: 389 ≥15 y: NA All: NA	1, 2
	Bandim, Guinea-Bissau [56]	2011	10^b^	0–1 y: 0 2–4 y: 53 5–14 y: 18 <15 y: 20 ≥15 y: 4 All: 10	1, 2
	Nioko, Burkina Faso [56]	2012	104^b^	0–1 y: 0 2–4 y: 251 5–14 y: 315 <15 y: 227 ≥15 y: 0 All: 104	1, 2
	Polesgo, Burkina Faso [56]	2012	383^b^	0–1 y: 0 2–4 y: 1890 5–14 y: 485 <15 y: 719 ≥15 y: 107 All: 383	1, 2
	Nanoro, Burkina Faso [49]	2013		<5 y: 224^b^ 5–15 y: 273^b^	1, 2, 3, 4
Asia					
Southern Asia	Dhaka, Bangladesh [57]	2003	280^b^	<5 y: 1600^b^ ≥5 y: 100^b^	2

Abbreviation: NA, not applicable; y, years.

^a^Multipliers used: (1) healthcare facility (eligible participants not seeking care at study clinic); (2) enrollment (eligible participants did not have a blood culture collected); (3) test sensitivity; (4) seasonality (surveillance period).

^b^Per 100 000 person-years observed.

1. Healthcare facility: eligible participants not seeking care at study clinic (16 [94.1%]);
2. Enrollment: eligible participants did not have a blood culture collected (15 [88.2%]);
3. Test sensitivity: an adjustment factor for the sensitivity of blood cultures, most often 2 times or 50% sensitivity (4 [23.5%]);
4. Seasonality: length of surveillance period (2 [11.8%]).

**Figure 4.**
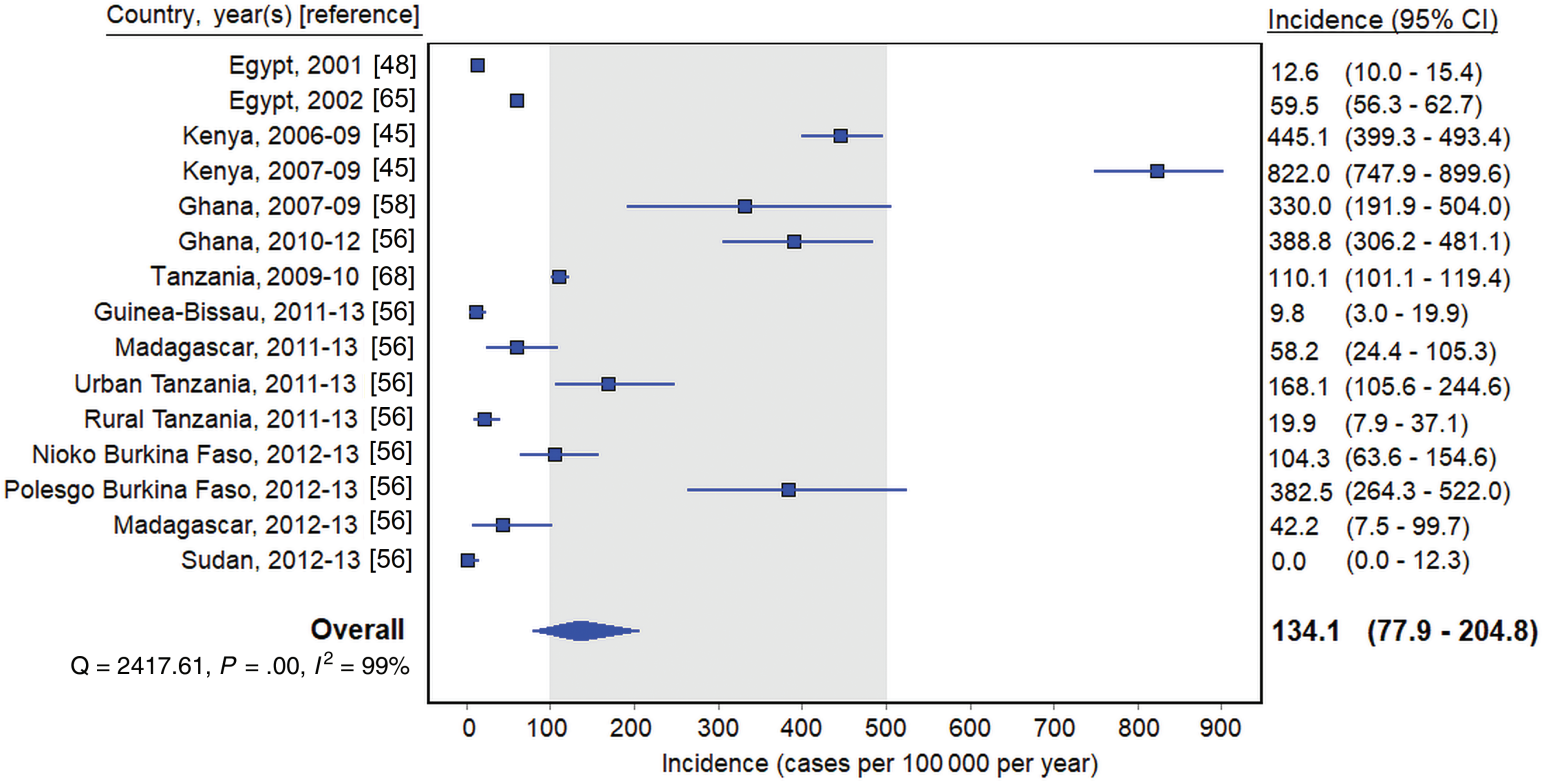
Typhoid incidence estimates among multiplier studies in Africa, 1954–2018. Gray shading indicates 100–500 per 100 000 per year. Abbreviation: CI, confidence interval.

In contrast to incidence studies without multipliers, the majority (11 [64.7%]) reporting an estimated incidence with a multiplier were recent, collecting data from 2010 through 2014. The overall pooled incidence estimate from multiplier studies was 141.8 (95% CI, 85.3–212.2) typhoid cases per 100 000 per year. All but 1 of the multiplier study sites were located in Africa [57]. Removing the site located in Asia, the pooled estimate of multiplier studies located in Africa was 134.1 (95% CI, 77.9–204.8) per 100 000 per year (Figure 4). Typhoid incidence varied between sites and countries, with no cases reported in East Wad Medani, Sudan [56], and 882.0 typhoid cases per 100 000 per year in Kibera, Kenya [45]. One study was excluded from the multiplier pooled estimate calculations because it did not report the population under surveillance, only the incidence by age group [49]. Heterogeneity was significant in each pooled estimate (*I*^2^ > 98%; *P* < .001, Cochran Q test).

## DISCUSSION

We performed a systematic review and meta-analysis of blood culture–confirmed typhoid fever incidence. Although an encouraging number of typhoid incidence studies meeting criteria for inclusion have been published since 2000, a substantial proportion had data collection periods much earlier and as long ago as 1954. When examined by time and region, there appears to be a secular trend of lower incidence in more recent studies. This is consistent with other work suggesting declining typhoid fever incidence worldwide [8].

A number of studies included in this review collected typhoid incidence data from the same location over multiple years of surveillance. While some locations such as Alexandria, Egypt [69], recorded stable typhoid incidence between years of surveillance, significant changes in incidence were recorded at others such as Eastern Transvaal, South Africa [52, 53], and Kolkata, India [66, 67]. As none of the studies included in our systematic review were done during recognized typhoid fever epidemics, the multiyear studies illustrate the considerable variation in typhoid incidence that may occur between years at the same site considered to have endemic disease.

In addition to variation of endemic typhoid fever in time at the same location, our systematic review illustrates the considerable variation in endemic typhoid incidence that may occur between sites within the same country, between countries within a region, and between regions. This serves as a reminder that limited typhoid surveillance data can be misleading and that decisions for typhoid vaccine introduction should be based not only on surveillance data but also be informed by the occurrence of risk factors for typhoid transmission such as unsafe water and unimproved sanitation facilities.

Sites in the Asia area have long been recognized as having high typhoid incidence [73]. However, some considered typhoid fever to be uncommon in the Africa area as recently as 2008 [74]. The substantial number of new typhoid fever incidence estimates from sites in Africa since earlier reviews [2, 8, 19] confirms that some African sites record high typhoid fever incidence [56] and provides evidence that TCV use should be considered beyond Asia.

We demonstrate that eligible contemporary typhoid fever incidence studies were not available for some areas and regions. Notably, no eligible studies were identified from Oceania despite the major typhoid fever problem that is recognized in some Pacific islands [75]. Studies were also lacking from high-income countries, that rely on robust national surveillance systems to monitor typhoid fever. However, typhoid fever incidence is almost universally low in high-income countries, is often travel-associated, and likely contributes little to total global cases [76]. While increasingly sophisticated approaches to modeling typhoid fever incidence provide a means of estimating typhoid fever incidence in locations where data are lacking [2, 19, 20], we sought to focus on systematic presentation of primary data and summary values across eligible studies.

Our quality assessment identified a number of concerns with published typhoid fever incidence studies. We rated 5 studies as high quality, thus classifying the majority as of moderate or low quality. Our data were also limited by the significant heterogeneity, but given the variation in location, inclusion ages, study design, and dates, this was to be expected. Our search strategy may not have identified negative studies from sites with low typhoid incidence.

A number of factors drove downgrading of study quality. Multiplier studies have been increasingly used to estimate typhoid fever incidence for cost and logistical reasons in recent years [48]. Such studies are less expensive and less logistically complex than population-based active surveillance studies and underpin considerable recent progress in available typhoid fever incidence data. However, the multiplier method has not been validated in the context of population-based surveillance and is associated with greater uncertainty introduced at each stage of correction for underascertainment. Furthermore, we identified considerable variation in the types of multipliers that were used. We recommend that consensus be reached on standardization of multiplier selection for future work to ensure that incidence estimates derived from multiplier studies are directly comparable.

We also found that patient selection may have introduced bias in a large number of studies. The control arms of vaccine trials remain an important source of data on typhoid fever incidence. However, it must be recognized that vaccine trials are likely to be preferentially done in areas of high typhoid incidence to increase statistical power. Furthermore, a number of studies were done in specific age groups or communities, and resulting incidence estimates may not reflect disease in all age groups or the wider population. We also identified variation in who received blood or bone marrow cultures in studies, which may have introduced bias, and blood culture volume adequacy and the proportion of blood cultures contaminated were rarely reported.

While there are concerns for bias, we describe an overall pattern of declining incidence among included studies, with variation in typhoid incidence between surveillance years at the same site and also variation in place within countries and regions. These observations support the recognition of heterogeneity in endemic typhoid fever in both time and place. They also underscore the importance of considering both surveillance data and typhoid fever risk factors or “receptivity” when making vaccine introduction decisions.

We encourage ongoing efforts to generate more contemporary estimates of typhoid incidence, focusing on areas and regions where data are lacking, including Oceania and many parts of Africa. We also identify the need to develop a consensus standard on the types of adjustments that should be made in multiplier studies. This systematic review serves as a resource for site selection for future incidence studies, as a data source for typhoid fever modeling efforts, and in policy decisions for typhoid fever control.

## Supplementary Data

Supplementary materials are available at *Clinical Infectious Diseases* online. Consisting of data provided by the authors to benefit the reader, the posted materials are not copyedited and are the sole responsibility of the authors, so questions or comments should be addressed to the corresponding author.

ciy1094_suppl_Supplementary_Data

ciy1094_suppl_Supplementary_Appendix_A-B

## Notes

***Author contributions.*** J. A. C. conceived the study. J. A. C. and C. Y. H. developed the protocol and search strategy, and performed the title and abstract review. J. A. C. and C. S. M. reviewed full-text articles and abstracted the data. C. S. M. performed the analysis, created the tables and figures, and wrote the first draft of the manuscript. J. A. C. provided major revisions and comments to the manuscript. All authors reviewed the manuscript for final revisions before submission.

***Acknowledgments.*** The authors thank Dr Richard German, Divisional Librarian, Division of Health Sciences Library, University of Otago, for assistance with development of the search strategy.

***Financial support.*** This publication is based on research funded in part by a grant from the Bill & Melinda Gates Foundation (OPP1151153). J. A. C., C. Y. H., and C. S. M. received support from BMGF grant OPP1151153. J. A. C. also received support from BMGF (grant numbers OPP1125993 and OPP1158210), the US National Institutes of Health (grant number R01AI121378), and the New Zealand Health Research Council through the e-ASIA Joint Research Program (grant number 16/697).

***Supplement sponsorship.*** This supplement is sponsored by the Center for Vaccine Development and Global Health (CVD) at the University of Maryland School of Medicine.

***Potential conflicts of interest.*** All authors: No reported conﬂicts. All authors have submitted the ICMJE Form for Disclosure of Potential Conﬂicts of Interest. Conﬂicts that the editors consider relevant to the content of the manuscript have been disclosed.
