# Supplementary material for: Global Typhoid Fever Incidence: A Systematic Review and Meta-analysis
Source: Clin Infect Dis. 2019 Mar 7;68(Suppl 2):S105–16. doi: 10.1093/cid/ciy1094 (PMC6405273; doi:10.1093/cid/ciy1094)
Supplement: ciy1094_suppl_Supplementary_Appendix_A-B [file ciy1094_suppl_supplementary_appendix_a-b.docx]

**APPENDIX**

**A - *Search strategy***

*Ovid search* (completed 19/1/2018)

1. typhoid-paratyphoid vaccine*. [mp=ti, ot, ab, sh, hw, kw, tn, dm, mf, dv, fx, nm, kf, px, rx, ui, sy]
2. typhoid.mp. [mp=ti, ot, ab, sh, hw, kw, tn, dm, mf, dv, fx, nm, kf, px, rx, ui, sy]
3. salmonella typhi.mp. [mp=ti, ot, ab, sh, hw, kw, tn, dm, mf, dv, fx, nm, kf, px, rx, ui, sy]
4. salmonella enterica serovar* typhi.mp. [mp=ti, ot, ab, sh, hw, kw, tn, dm, mf, dv, fx, nm, kf,
px, rx, ui, sy]
5. enteric fever.mp. [mp=ti, ot, ab, sh, hw, kw, tn, dm, mf, dv, fx, nm, kf, px, rx, ui, sy]
6. remove duplicates from 1
7. remove duplicates from 4
8. remove duplicates from 5
9. 2 or 4 or 6 or 7 or 8
10. incidence*.mp. [mp=ti, ot, ab, sh, hw, kw, tn, dm, mf, dv, fx, nm, kf, px, rx, ui, sy]
11. (incidence* adj4 (typhoid or salmonella typhi)).mp. [mp=ti, ot, ab, sh, hw, kw, tn, dm, mf, dv, fx, nm, kf, px, rx, ui, sy]
12. (burden* adj4 (typhoid or salmonella typhi)).mp. [mp=ti, ot, ab, sh, hw, kw, tn, dm, mf, dv,
fx, nm, kf, px, rx, ui, sy]
13. (culture* adj4 (typhoid or salmonella typhi)).mp. [mp=ti, ot, ab, sh, hw, kw, tn, dm, mf, dv,
fx, nm, kf, px, rx, ui, sy]
14. (attack rate* adj4 (typhoid or salmonella typhi)).mp. [mp=ti, ot, ab, sh, hw, kw, tn, dm, mf,
dv, fx, nm, kf, px, rx, ui, sy]
15. (case* adj4 (typhoid or salmonella typhi)).mp. [mp=ti, ot, ab, sh, hw, kw, tn, dm, mf, dv, fx, nm, kf, px, rx, ui, sy]
16. efficac*.mp. [mp=ti, ot, ab, sh, hw, kw, tn, dm, mf, dv, fx, nm, kf, px, rx, ui, sy]
17. protective effect*.mp. [mp=ti, ot, ab, sh, hw, kw, tn, dm, mf, dv, fx, nm, kf, px, rx, ui, sy]
18. remove duplicates from 11
19. remove duplicates from 12
20. remove duplicates from 13
21. remove duplicates from 14
22. remove duplicates from 15
23. 10 or 16 or 17 or 18 or 19 or 20 or 21 or 22
24. 9 and 23
25. (exp animals/ or nonhuman/) not exp human/
26. 24 not 25
27. limit 26 to (meta analysis or “systematic review”) [Limit not valid in CCTR,Ovid
MEDLINE(R),Ovid MEDLINE(R) Daily Update; records were retained]
28. limit 26 to (meta analysis or systematic reviews) [Limit not valid in CCTR,Embase; records
were retained]
29. 26 not (27 or 28)
30. Remove duplicates from 29

*Scopus Search* (completed 22/1/2018)
( ( TITLE-ABS-KEY ( typhoid-paratyphoid AND vaccine* ) ) OR ( TITLE-ABS-KEY ( typhoid ) ) OR (TITLE-ABS-KEY ( salmonella AND typhi ) ) OR ( TITLE-ABS-KEY ( salmonella AND enterica AND serovar* AND typhi ) ) OR ( TITLE-ABS-KEY ( enteric AND fever ) ) ) AND ( ( TITLE-ABS-KEY (incidence* ) ) OR ( TITLE-ABS-KEY ( ( incidence* W/4 typhoid ) OR ( incidence* W/4 salmonella AND typhi ) ) ) OR ( TITLE-ABS-KEY ( ( burden* W/4 typhoid ) OR ( burden* W/4 salmonella AND typhi ) ) ) OR ( TITLE-ABS-KEY ( ( culture* W/4 typhoid ) OR ( culture* W/4 salmonella AND typhi) ) ) OR ( TITLE-ABS-KEY ( ( attack AND rate* W/4 typhoid ) OR ( attack AND rate* W/4 salmonella AND typhi ) ) ) OR ( TITLE-ABS-KEY ( ( case* W/4 typhoid ) OR ( case* W/4 salmonella AND typhi ) ) ) OR ( TITLE-ABS-KEY ( efficac* ) ) OR ( TITLE-ABS-KEY ( protective AND effect* ) ) ) AND ( EXCLUDE ( EXACTKEYWORD , "Nonhuman" ) )

*
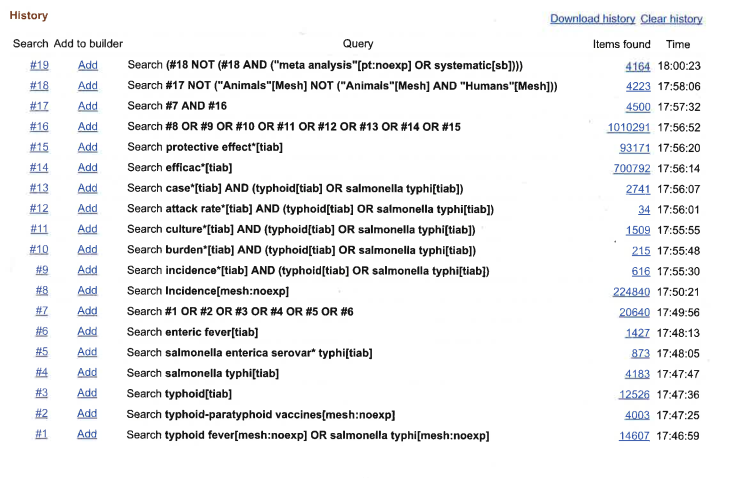
PubMed search* (completed 22/1/2018)

**B - *Quality assessment***

| **Author, Year** | Patient flow/study design (e.g. active surveillance, multiplier studies) introduced bias? (H/M/L/U) | Appropriately randomized and concealed allocation? (Y/N/U/I) | Likelihood that patient selection could have introduced bias (age/community)? (H/M/L/U) | Reporting blood culture volume adequate and contamination? (H/M/L/U) | Blind outcome assessors from knowledge of which intervention a participant received? (Y/N/U/I) | Bias of clinical case definition received a blood or bone marrow culture? (H/M/L/U) | **Overall assessment of quality (H/M/L)** |
| --- | --- | --- | --- | --- | --- | --- | --- |
|  | Selection and performance | | | Reference standard | | |  |
| Acharya, I.,1987 | L | Y | L | U | U | L | **H** |
| Breiman, R.,2012 | L | I | L | U | I | L | **H** |
| Lin, F.,2000 | L | I | L | U | I | L | **H** |
| Naheed, A.,2010 | L | I | L | U | I | L | **H** |
| Sinha, A.,1999 | L | I | L | U | I | L | **H** |
| Brooks, W.,2005 | L | I | L | U | I | M | **M** |
| Crump, J.,2003 | M | I | L | U | I | M | **M** |
| Guiraud, I.,2017 | L | I | M | U | I | L | **M** |
| Marks, F.,2017 | M | I | L | U | I | L | **M** |
| Ochiai, R.,2008 | L | I | M | U | I | M | **M** |
| Owais, A.,2010 | L | I | H | U | I | L | **M** |
| Punjabi, N.,2013 | M | I | L | U | I | M | **M** |
| Srikantiah, P.,2006 | M | I | L | U | I | L | **M** |
| Sur, D.,2006 | L | I | L | U | I | M | **M** |
| Sur, D.,2009 | L | Y | M | U | U | M | **M** |
| Thriemer, K.,2012 | M | I | L | L | I | L | **M** |
| Yang, H.,2001 | L | Y | M | U | Y | L | **M** |
| Black, R.,1990 | L | Y | M | U | Y | H | **L** |
| Chuttani, C.,1977 | L | Y | H | U | U | M | **L** |
| Hejfec, L.,1968 | L | U | M | U | U | M | **L** |
| Hejfec, L.,1969 | L | U | M | U | U | M | **L** |
| Khan, M.,2012 | M | Y | M | U | Y | M | **L** |
| Klugman, K.,1987 | M | Y | H | U | Y | M | **L** |
| Klugman, K.,1996 | M | Y | H | U | Y | M | **L** |
| Levine, M.,1999 | M | Y | H | U | Y | H | **L** |
| Lin, F.,2001 | L | Y | H | U | Y | L | **L** |
| Mitra, M.,2016 | L | U | M | U | U | M | **L** |
| Nielsen, M.,2012 | M | I | M | U | I | L | **L** |
| Siddiqui, F.,2006 | L | I | M | U | I | M | **L** |
| Simanjuntak, C.,1991 | L | Y | H | U | Y | M | **L** |
| Wahdan, M.,1982 | L | Y | H | U | Y | M | **L** |
| Wang, Z. 1997 | M | Y | M | U | U | M | **L** |
| Yugoslav TC,1962 | M | Y | M | U | Y | M | **L** |

Low (L), moderate (M), High (H), Yes (Y), No (N), Unknown (U), Inapplicable (I)

Category was considered with greater weight
